# Supplementary material for: Celiac disease and COVID-19 in adults: A systematic review
Source: PLoS One. 2023 May 16;18(5):e0285880. doi: 10.1371/journal.pone.0285880 (PMC10187909; doi:10.1371/journal.pone.0285880)
Supplement: S1 File — (DOCX) [file pone.0285880.s001.docx]

**Supplemental Table 1** Detailed search terms in the databases (up to 11/17/2022)

| **Database** | **Search term** |
| --- | --- |
| PUBMED  **(n = 75)** | ("Celiac Disease"[mesh] OR "Celiac Disease"[tiab] OR "Gluten Enteropathy"[tiab] OR "Gluten Enteropathies"[tiab] OR "Gluten Sensitive Enteropathy"[tiab] OR "Nontropical Sprue"[tiab] OR "Celiac Sprue"[tiab]) AND ("covid-19"[mesh] OR "covid-19"[tiab] OR "COVID 19"[tiab] OR "SARS CoV 2 Infection"[tiab] OR "2019 Novel Coronavirus Disease"[tiab] OR "2019 Novel Coronavirus Infection"[tiab] OR "2019 nCoV Disease"[tiab] OR "COVID 19 Virus Infection"[tiab] OR "Coronavirus Disease 2019"[tiab] OR "Coronavirus Disease 19"[tiab] OR "Severe Acute Respiratory Syndrome Coronavirus 2 Infection"[tiab] OR "SARS Coronavirus 2 Infection"[tiab] OR "COVID 19 Virus Disease"[tiab] OR "2019 nCoV Infection"[tiab] OR "COVID19"[tiab] OR "COVID-19 Pandemic"[tiab] OR "COVID 19 Pandemic"[tiab] OR "COVID-19 Pandemics"[tiab] OR "SARS-CoV-2 Infection"[tiab] OR "SARS-CoV-2 Infections"[tiab] OR "2019-nCoV Disease"[tiab] OR "2019-nCoV Diseases"[tiab] OR "COVID-19 Virus Infection"[tiab] OR "COVID-19 Virus Infections"[tiab] OR "Coronavirus Disease-19"[tiab] OR "COVID-19 Virus Disease"[tiab] OR "2019-nCoV Infection"[tiab] OR "2019-nCoV Infections"[tiab]) |
| SCOPUS  )**n=176)** | ( TITLE-ABS-KEY ( "Celiac Disease" OR "Gluten Enteropathy" OR "Gluten Enteropathies" OR "Gluten Sensitive Enteropathy" OR "Nontropical Sprue" OR "Celiac Sprue" ) AND TITLE-ABS-KEY ( "covid-19" OR "COVID 19" OR "SARS CoV 2 Infection" OR "2019 Novel Coronavirus Disease" OR "2019 Novel Coronavirus Infection" OR "2019 nCoV Disease" OR "COVID 19 Virus Infection" OR "Coronavirus Disease 2019" OR "Coronavirus Disease 19" OR "Severe Acute Respiratory Syndrome Coronavirus 2 Infection" OR "SARS Coronavirus 2 Infection" OR "COVID 19 Virus Disease" OR "2019 nCoV Infection" OR "COVID19" OR "COVID-19 Pandemic" OR "COVID 19 Pandemic" OR "COVID-19 Pandemics" OR "SARS-CoV-2 Infection" OR "SARS-CoV-2 Infections" OR "2019-nCoV Disease" OR "2019-nCoV Diseases" OR "COVID-19 Virus Infection" OR "COVID-19 Virus Infections" OR "Coronavirus Disease-19" OR "COVID-19 Virus Disease" OR "2019-nCoV Infection" OR "2019-nCoV Infections" ) ) |
| EMBASE  **(n=258)** | ('celiac disease'/exp OR 'celiac disease') AND ('coronavirus disease 2019'/exp OR 'coronavirus disease 2019') |
