# Supplementary material for: Celiac disease and COVID-19 in adults: A systematic review
Source: PLoS One. 2023 May 16;18(5):e0285880. doi: 10.1371/journal.pone.0285880 (PMC10187909; doi:10.1371/journal.pone.0285880)
Supplement: S1 Table — (DOCX) [file pone.0285880.s003.docx]

S1. Quality Assessment

| Author (ref.) | Selection  1 2 3 4 | | | | Comparability | Outcome  1 2 3 | | | Total score | Total Quality |
| --- | --- | --- | --- | --- | --- | --- | --- | --- | --- | --- |
| Al Hayek et al. |  |  | * |  | * | * | * |  | 4 | satisfactory |
| Elli et al. | * |  |  |  | ** |  | * |  | 4 | satisfactory |
| Falcomer et al. |  | * | * |  |  |  |  |  | 2 | unsatisfactory |
| Gasbarrini et al. | * |  | * |  | * |  | * | * | 5 | satisfactory |
| Gholam-Mostafaei et al. | * |  | * |  | ** |  | * | * | 6 | Good |
| Gokden et al. | * |  | * |  | ** |  | * |  | 5 | satisfactory |
| Greco et al. | * |  | * |  | * |  | * |  | 4 | satisfactory |
| Hadi et al. | * | * | * |  | ** | * | * | * | 8 | excellent |
| Ibsen et al. | * | * | * | * | * | * | * |  | 7 | Good |
| Lebwohl et al. | * | * | * |  | * | * | * | * | 7 | Good |
| Li et al. | * | * | * |  | * | * | * | * | 7 | Good |
| Mehtab et al. | * |  | * |  | * | * |  |  | 4 | satisfactory |
| M¨oller et al | * |  | * |  | * |  |  |  | 3 | unsatisfactory |
| Schiepatti et al. | * | * | * |  | ** | * | * | * | 8 | excellent |

Scores:

Excellent: 8-9 points

Good: 6-7 points

Satisfactory: 4-5 points

Unsatisfactory: 0-3 points
